# Quantitative single-molecule microscopy reveals that CENP-A^Cnp1^ deposition occurs during G2 in fission yeast

**DOI:** 10.1098/rsob.120078

**Published:** 2012-07

**Authors:** David Lando, Ulrike Endesfelder, Harald Berger, Lakxmi Subramanian, Paul D. Dunne, James McColl, David Klenerman, Antony M. Carr, Markus Sauer, Robin C. Allshire, Mike Heilemann, Ernest D. Laue

**Affiliations:** 1 Department of Biochemistry, University of Cambridge, Cambridge CB2 1EW, UK; 2 Department of Chemistry, University of Cambridge, Cambridge CB2 1EW, UK; 3 Department of Biotechnology and Biophysics, Julius-Maximilians-University Würzburg, Am Hubland, 97074 Wurzburg, Germany; 4 Wellcome Trust Centre for Cell Biology and Institute of Cell Biology, The University of Edinburgh, Edinburgh EH9 3JR, UK; 5 Genome Damage and Stability Centre, University of Sussex, Falmer, Brighton BN1 9RQ, UK

**Keywords:** CENP-A, centromere, single-molecule microscopy, fission yeast

## Abstract

The inheritance of the histone H3 variant CENP-A in nucleosomes at centromeres following DNA replication is mediated by an epigenetic mechanism. To understand the process of epigenetic inheritance, or propagation of histones and histone variants, as nucleosomes are disassembled and reassembled in living eukaryotic cells, we have explored the feasibility of exploiting photo-activated localization microscopy (PALM). PALM of single molecules in living cells has the potential to reveal new concepts in cell biology, providing insights into stochastic variation in cellular states. However, thus far, its use has been limited to studies in bacteria or to processes occurring near the surface of eukaryotic cells. With PALM, one literally observes and ‘counts’ individual molecules in cells one-by-one and this allows the recording of images with a resolution higher than that determined by the diffraction of light (the so-called super-resolution microscopy). Here, we investigate the use of different fluorophores and develop procedures to count the centromere-specific histone H3 variant CENP-A^Cnp1^ with single-molecule sensitivity in fission yeast (*Schizosaccharomyces pombe*). The results obtained are validated by and compared with ChIP-seq analyses. Using this approach, CENP-A^Cnp1^ levels at fission yeast (*S. pombe*) centromeres were followed as they change during the cell cycle. Our measurements show that CENP-A^Cnp1^ is deposited solely during the G2 phase of the cell cycle.

## 2. Introduction

All centromeres are characterized by the presence of unusual centromere-specific nucleosomes in which histone H3 is replaced by the histone H3 variant CENP-A. In species with regional centromeres, it is clear that the establishment of CENP-A chromatin at particular locations is epigenetically determined. This CENP-A chromatin serves as a foundation for the assembly of kinetochores, the complex protein machinery that is required for microtubule attachment and accurate chromosome segregation [1,2].

When centromeric DNA is duplicated during early S phase [3], CENP-A-containing nucleosomes are segregated into the two daughter strands. Thus, each new centromere has only half the previous number of CENP-A nucleosomes, which must be replenished prior to the next round of replication. The underlying molecular mechanism by which this occurs and how these events are coordinated with the cell cycle remains unclear. Studies in the budding yeast *Saccharomyces cerevisiae* have shown that CENP-A^Cse4^ deposition occurs during S phase either in conjunction with or just after centromere replication [4]. By contrast, evidence from *Drosophila* embryos [5], *Drosophila* S2 cells [6] and human cells [7] shows that CENP-A (Centromere IDentifier, CID, in flies) is not replenished during DNA replication in S phase, but is instead deposited onto centromeric chromatin during anaphase, metaphase and the late stages of mitosis/early G1, respectively. This suggests that for a significant part of the cell cycle, from S phase through to the end of mitosis, metazoan centromeres contain half the number of CENP-A nucleosomes than are present from G1 until centromere replication. Indeed, recent analyses suggest that in human cells, histone H3.3 is deposited in S phase as a ‘placeholder’, which is replaced with CENP-A in G1 [8]. Intriguingly, in the fission yeast *Schizosaccharomyces pombe*, deposition of CENP-A^Cnp1^ has been reported to occur both in S phase and in G2 before cells undergo mitosis [9,10].

In recent years, the development of new light microscopy methods that allow imaging beyond the diffraction-limit of light (super-resolution) has paved the way for visualizing cellular structures with near-molecular resolution [11]. Methods such as photo-activated localization microscopy (PALM) have proved powerful tools for studying the organization of single protein molecules in bacteria [12] and in eukaryotic cells [13]. Despite these improvements in resolution, the use of PALM or other similar techniques to precisely count and quantify molecules is lacking. Here, we have investigated the feasibility of using PALM to study the deposition of CENP-A^Cnp1^ at *S. pombe* centromeres during the cell cycle at single-molecule sensitivity. Our aim was to compare the number of CENP-A^Cnp1^-containing nucleosomes that are present during the different phases of the cell cycle and to understand when CENP-A^Cnp1^ molecules are replenished.

## 3. Results and discussion

### 3.1. Super-resolution photo-activated localization microscopy imaging of CENP-A^Cnp1^

To image single molecules of CENP-A^Cnp1^ at super-resolution using PALM, we tested different fluorophores and tagged the endogenous CENP-A^Cnp1^ encoding gene with the photo-convertible mEos2 [14]. Although tagging CENP-A^Cnp1^ at the C-terminus with green fluorescent protein (GFP) has been previously reported to cause growth retardation, tagging at the N-terminus at the authentic chromosomal locus resulted in cells that have near wild-type viability. Moreover, the expression level of GFP-CENP-A^Cnp1^ was comparable with that of the wild-type protein [9]. We confirmed that CENP-A^Cnp1^ tagged with mEos2 at the N-terminus did not lead to slower growth or lower viability when compared with either an untagged *cnp1^+^* wild-type strain or with other similar strains expressing CENP-A^Cnp1^ tagged with GFP (see the electronic supplementary material, figure S1). Moreover, chromatin immunoprecipitation (ChIP) showed that similar levels of CENP-A^Cnp1^ are detected at centromeres (see later text). In addition, during the image analysis, we used a number of approaches to confirm that the cells were healthy and had no apparent defects in chromosome segregation (see the electronic supplementary material, figure S2). We conclude that the mEos2–CENP-A^Cnp1^ fusion protein is essentially fully functional.

**Figure 1.**
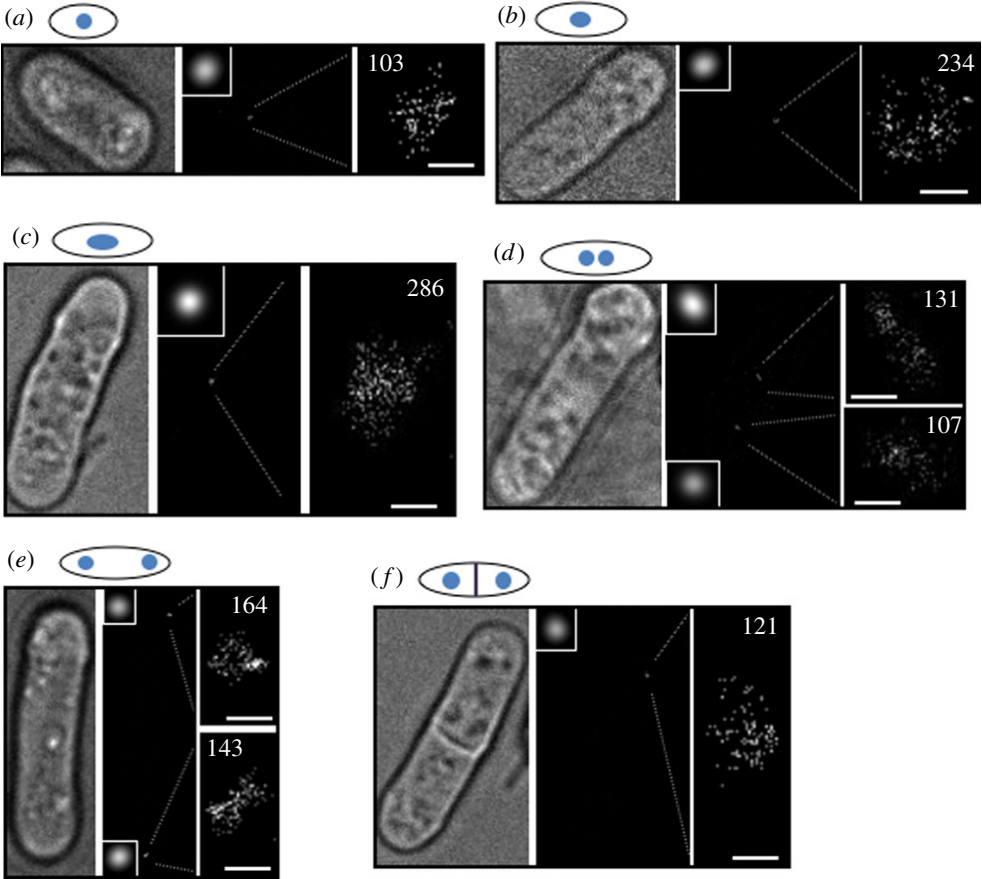
Photo-activated localization microscopy imaging of *Schizosaccharomyces pombe* mEos2–CENP-A^Cnp1^ at different stages of the cell cycle. Representative images from (*a*) early G2, (*b*) mid-G2, (*c*) late G2, (*d*) early and (*e*) late anaphase of mitosis and (*f*) S phase (after septum formation) DL56 cells. The left panel is a white light transmission image, whereas the middle panel is a reconstructed PALM image with a magnified diffraction-limited image of the CENP-A^Cnp1^ cluster (inset). The right panel is a magnified PALM image of the CENP-A^Cnp1^ cluster with the number of localizations indicated. Note that only one cluster from (*f*) was in focus. (*a*–*f*) Scale bars, 200 nm.

**Figure 2.**
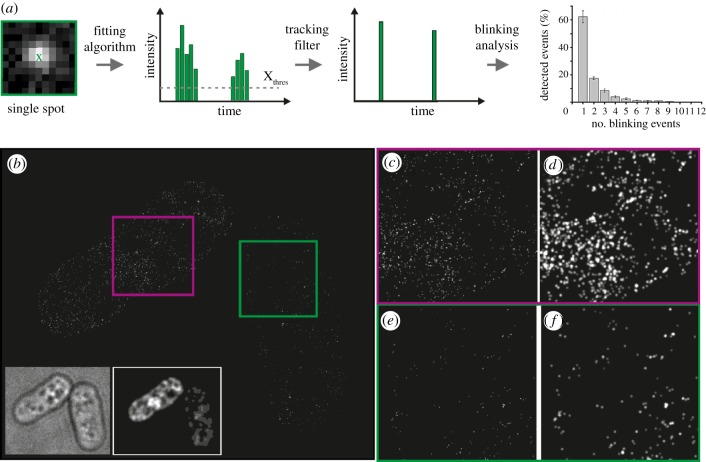
Counting isolated/free mEos2 fluorescent protein molecules using PALM. (*a*) Schematic outlining the process used to determine the average number of blinking events for single mEos2 fluorophores. Using data derived from single-molecule PALM analysis of *Schizosaccharomyces pombe* expressing low levels of mEos2, we found an average of 1.98 localizations (blinking events) per mEos2 molecule. (*b*) PALM image of free mEos2 fluorescent protein expressed from the nmt81 promoter (strain DL41). Individual cells show different expression levels of the mEos2 protein (insets: bright field, left; diffraction-limited fluorescence images, right). Magnified view of each cell with a high expression level (*c*,*d*, purple box) and a low expression level (*e*,*f*, green box) of mEos2 fluorophores; (*c*,*e*) images after Kalman filtering; (*d*,*f*) images that were intensity-blurred with the experimental resolution calculated from the number of photons for each single-molecule localization.

To acquire a super-resolution PALM image, we photo-converted the mEos2 fluorophore with a sufficiently low-intensity UV laser so as to excite and image only one individual mEos2–CENP-A^Cnp1^ molecule at a time. This allowed us to generate an image of CENP-A^Cnp1^ that has much superior resolution to that obtained with conventional diffraction-limited imaging (figure 1). An analysis of mEos2 images revealed that a resolution of 21 nm could be achieved with our PALM set-up (see the electronic supplementary material, figure S3). However, because of the very dense packing of CENP-A^Cnp1^ molecules, this would still not be sufficient to resolve individual proteins within the cluster if it were not for the fact that individual mEos2–CENP-A^Cnp1^ molecules are detected at different times in the PALM experiment. As we detect and identify only the location of one single molecule at a time, observation and counting of the number of CENP-A^Cnp1^ molecules are not restricted by the spatial resolution because an adjacent molecule will, in general, be detected at a substantially different time during the experiment (described later in more detail, and also in figure 2 and the electronic supplementary information, figure S4). Compared with conventional fluorescence microscopy (resolution greater than 200 nm), our strategy enables the location of individual CENP-A^Cnp1^ molecules in the clustered centromeres, which remain associated through much of the cell cycle in *S. pombe* [15].

### 3.2. Counting CENP-A^Cnp1^ molecules

Given our success in visualizing individual CENP-A^Cnp1^ molecules using PALM, we next investigated whether it is possible to quantify the numbers of imaged molecules per CENP-A^Cnp1^ cluster. A key factor in quantitative PALM imaging is to ensure that only one molecule is observed at a given time; this requires photo-activation/conversion with sufficiently low-intensity light followed by imaging with high-intensity activating light until the fluorophore bleaches, at which point it cannot be reactivated. Another factor that can confound quantification is that a single molecule is detected typically over more than one image. Furthermore, after activation, some fluorophores transiently switch off and then later switch back on again—a phenomenon known as blinking, which can occur multiple times. These problems, when coupled to imprecise localization, can lead to subsequent re-counting of the same fluorophore molecule. Previous studies have shown that mEos2 does blink [14,16], and that this rate can change under different environmental conditions [17]. A possible strategy to account for these factors and to extract robust information on protein numbers is to combine PALM imaging with spatial correlation analysis of single-molecule distributions [18]. However, this approach requires a sufficient number of clustered distributions in order to extract quantitative information from the correlation function, and thus is not suited to the analysis of close adjacent molecules such as are present in the single CENP-A^Cnp1^ cluster in a cell. Here, we adapted and extended an algorithm that groups multiple localizations of a single fluorophore and accounts for on–off blinking [19].

In the first part of the analysis, the software identifies peaks in individual image frames having a fluorescence intensity above a certain threshold, and fits a Gaussian peak to each of these to determine the coordinates of single mEos2 fluorophores (fitting algorithm, figure 2*a*). Then, to account for different mEos2 fluorophore ‘on-times’, the software uses a Kalman algorithm to scan and group together single fluorophore molecules that were identified (with an initial distance of 40 nm of each other in the *x–y* plane) in sequential frames (tracking filter, figure 2*a*). The coordinates of the mEos2–CENP-A^Cnp1^ localizations identified after Kalman filtering (and their intensities) are finally used to reconstruct synthetic PALM images (as shown in figure 1). (Note that the resulting synthetic images lack most background noise, because this is not fitted during the image analysis. Further details of the software and algorithms used can be found in the electronic supplementary material, figure S4 and section ‘Material and methods’.) To account for mEos2 on–off blinking, we calibrated our PALM imaging experiments using untagged mEos2 expressed in *S. pombe* cells grown in identical media and conditions (figure 2*b–f*). To reduce the occurrence of overlapping mEos2 molecules, we analysed cells expressing low amounts of mEos2 and found that only one single-molecule localization is detected for most individual mEos2 molecules (approx. 62%; figure 2*a*). However, approximately 18 per cent of the mEos2 molecules blink twice with some blinking up to nine times (approx. 1%). On average, this means that each mEos2 molecule blinks twice (figure 2*a*). In an additional control experiment, we verified that in wild-type cells (i.e. not containing any mEos2) the number of false localizations found within regions with the same dimensions as a centromere cluster is less than one (see the electronic supplementary material, figure S3*b*). By calculating the blinking rate of mEos2 in *S. pombe* cells, we are therefore able to translate the number of single-molecule localizations detected in a PALM experiment into the actual number of mEos2 fluorophores. Thus, the 143 and 164 localizations detected by PALM imaging in the two clusters of a representative anaphase cell (figure 1*e*) corresponds to 72 and 82 molecules of CENP-A^Cnp1^ at each of the centromere clusters.

To assess the accuracy of counting mEos2-tagged proteins using PALM in this way, we tagged CENP-A^Cnp1^ with a second fluorophore, PAmCherry1 [20]. Although PAmCherry1 can be imaged in the same way as mEos2, it emits fewer photons, making the tagged protein localizations more difficult to distinguish from background noise. PAmCherry1, however, exhibits very little blinking in our hands (data not shown). Using PAmCherry1-tagged CENP-A^Cnp1^, we detected an average of 50 localizations (and thus molecules) at the centromere cluster in similar M/G1/early S phase cells (see the electronic supplementary material, figure S5). The detection of a somewhat lower number of PAmCherry1-tagged CENP-A^Cnp1^ localizations is expected because a higher proportion of these are likely to be classified as noise. This result validates our approach and the division by a factor of two that we use to convert mEos2 localizations into numbers of molecules. Although it blinks, we decided to work with mEos2 because it is brighter, which both increases the localization accuracy and reduces the number of falsely identified localizations (noise).

### 3.3. Comparison of CENP-A^Cnp1^ single-molecule counting with CENP-A^Cnp1^ nucleosome occupancy

**Figure 3.**
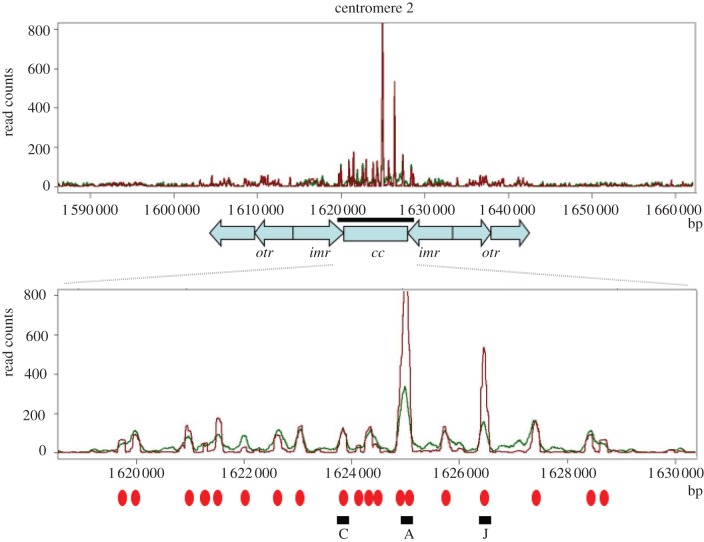
Distribution of CENP-A^Cnp1^ nucleosomes across the centromere from chromosome 2. Genome view of the centromere region showing ChIP-seq binding profiles of CENP-A^Cnp1^ from sonicated (green) and MNase-digested samples (red) isolated from the wild-type strain. Shown below is the magnified central domain region (black bar) with CENP-A^Cnp1^ nucleosome positions marked with red ovals. A, C and J indicate CENP-A^Cnp1^ nucleosome positions used for qPCR measurement (see the electronic supplementary material, figure S7), *cc* is the central core domain, *imr* the innermost repeats and *otr* the outer repeats. Data on the *y*-axis are normalized read counts, and the *x*-axis is genome position in base pairs.

To validate that we can count individual CENP-A^Cnp1^ molecules with reasonable accuracy using PALM, we determined the number of CENP-A^Cnp1^-containing nucleosomes at the three *S. pombe* centromeres using a different method. To determine a population average for the number of CENP-A^Cnp1^-containing nucleosomes across multiple cells, we used ChIP with anti-CENP-A^Cnp1^ sera followed by high-throughput DNA sequencing (ChIP-seq; figure 3 and the electronic supplementary material, figure S6). The profiles of both sonicated and micrococcal nuclease (MNase) digested chromatin revealed similar occupancies for CENP-A^Cnp1^ across the central kinetochore domain of all three centromeres, where CENP-A^Cnp1^ is known to be almost exclusively localized [21,22]. By digesting the DNA with MNase, and sequencing single-nucleosome-sized fragments of DNA using paired-end sequencing, we were able to show that the profiles reveal that the central domains of centromeres 1, 2 and 3 contain on average 21, 19 and 24 CENP-A^Cnp1^-containing nucleosomes, respectively. This indicates that a maximum of approximately 64 CENP-A^Cnp1^-containing nucleosomes are formed in each haploid cell, although not all of these nucleosome-binding positions are necessarily occupied in every cell at any one time.

The *in vivo* composition of CENP-A nucleosomes has been widely disputed (for a recent review, see Black & Cleveland [2]). The crystal structure of a human CENP-A-containing nucleosome shows that it contains two molecules of CENP-A [23], consistent with the structure of the CENP-A/H4 tetramer [24,25] and analysis of *Drosophila* CID mono-nucleosomes [26]. However, it has also been proposed that CENP-A can form hemisomes containing one copy each of CENP-A, H4, H2A and H2B [27]. Thus, the ChIP-seq experiments suggest that if the three centromeres are all fully occupied by approximately 20 nucleosomes, each containing two CENP-A^Cnp1^ subunits, we should expect a maximum total of approximately 120 CENP-A^Cnp1^ molecules (20 nucleosomes × two CENP-A^Cnp1^ molecules × three centromeres) at fission yeast centromeres per haploid (1N) genome (prior to S phase), and approximately 240 total (20 × 2 × 6) per diploid (2N) genome in G2. If, however, CENP-A were present as hemisomes, then we might expect approximately 60 CENP-A molecules per haploid (1N) genome, and approximately 120 per diploid (2N) genome. The numbers of CENP-A^Cnp1^ molecules that we counted using PALM for single haploid (1N) clusters in the anaphase cell in figure 1*e* were 72 and 82 molecules (mean 59, s.d. ± 17.8), i.e. somewhat more than half as many as the maximum total suggested by a ChIP-seq analysis.

Previous studies with similar fluorophores suggest that a certain fraction of fluorophore molecules are likely to remain dark or non-fluorescent (typically approx. 20%), and these cannot be excited and subsequently imaged [28,29]. It is likely, therefore, that counting proteins using PALM leads to an underestimate of the actual numbers of CENP-A^Cnp1^ molecules at the centromere. This suggests that CENP-A nucleosomes in fission yeast are more likely to contain two molecules of CENP-A^Cnp1^ because our measurements suggest that there are likely to be approximately 90 and 102 CENP-A^Cnp1^ molecules in 64 nucleosome positions in the anaphase cell shown in figure 1*e* (i.e. 72/0.8 and 82/0.8 molecules to correct for the 20% that remain dark or non-fluorescent). This is considerably more than the 64 CENP-A^Cnp1^ molecules we would expect if each position were occupied by a hemisome. It is also possible that not all the positions are occupied by a CENP-A^Cnp1^ nucleosome in every cell. Thus, the actual number of CENP-A^Cnp1^-containing nucleosomes could well be less than that predicted by the ChIP-seq analysis, which should represent the upper limit. The PALM experiments show that in different cells the number of CENP-A nucleosomes in M/G1/early S phase is variable, but less than 20/centromere, and suggest an average occupancy of approximately 58 per cent (59/0.8 × 100/128). However, we note that other explanations could account for the detection of lower than expected amounts of CENP-A^Cnp1^ at the centromere using PALM. For example, it has been suggested that different types of CENP-A nucleosomes (i.e. octasomes and hemisomes) might coexist in centromeric chromatin *in vivo* [2].

We tested whether the lower than expected amounts of CENP-A^Cnp1^ could be due to the mEos2 tag interfering with CENP-A^Cnp1^ deposition, leading to lower amounts of CENP-A^Cnp1^ at centromeres. Previously, we have shown that mutants that reduce the level of CENP-A^Cnp1^ protein at any of the three centromeres display a reciprocal increase in the levels of histone H3 within the central CENP-A^Cnp1^ domain [30]. More recently, using ChIP-seq analyses, we have found that CENP-A^Cnp1^ and histone H3 occupy exactly the same nucleosome-binding sites within the centromere (H. Berger & R. C. Allshire 2012, unpublished data). Thus, histone H3 levels at the centromere are inversely correlated with CENP-A^Cnp1^ occupancy. We determined the levels of CENP-A^Cnp1^ and histone H3 at three positions within the central domain of chromosome 2 in the mEos2–CENP-A^Cnp1^-tagged strain—C, A and J (see the electronic supplementary material, figure S7)—that represent positions of CENP-A^Cnp1^ nucleosomes in our CHIP-seq experiments (figure 3). Quantitative PCR (qPCR) analysis of CENP-A^Cnp1^ ChIP revealed an approximately 30–50% reduction in detected CENP-A^Cnp1^ at these three regions compared with the wild-type strain (see the electronic supplementary material, figure S7). This reduction was similar to that seen with an N-terminal-tagged GFP strain constructed by Takayama *et al*. [9]. Given that the antibody used in our experiments was raised to the amino terminus of CENP-A^Cnp1^, we suspected that the N-terminal tag may interfere to some degree with antibody binding, leading to reduced detection by ChIP–qPCR. This conclusion was supported by the histone H3 ChIP–qPCR analysis, which revealed similarly low levels of histone H3 in the mEos2-tagged, N-terminal GFP-tagged and wild-type strains (see the electronic supplementary material, figure S7). When taken together, the CENP-A^Cnp1^ and histone H3 ChIP experiments suggest, therefore, that the N-terminal mEos2 and GFP tags do not substantially alter the amount of CENP-A^Cnp1^ protein at centromeres. (This contrasts with the C-terminal GFP-tagged (S65T mutant) CENP-A^Cnp1^ strain [31], which exhibits clear growth defects and where Cnp1 deposition does appear to be compromised leading to the accumulation of histone H3 in place of CENP-A^Cnp1^—see the electronic supplementary material, figures S1 and S7.)

Our single-molecule PALM analysis of mEos2–CENP-A^Cnp1^ thus provides a minimum estimate, whereas the ChIP-seq nucleosome positioning data are suggestive of an upper estimate of the number of *S. pombe* centromeric nucleosomes that contain CENP-A^Cnp1^. Thus, our results reveal that at fission yeast centromeres the number of CENP-A^Cnp1^ nucleosomes (10–20) greatly exceeds the number of microtubule binding sites (2–4) [32], suggesting that microtubules might either randomly select CENP-A^Cnp1^ nucleosomes or there may exist a subset of elite CENP-A^Cnp1^ nucleosomes that are earmarked to assemble microtubule–kinetochore connections.

### 3.4. CENP-A^Cnp1^ is replenished in G2

**Figure 4.**
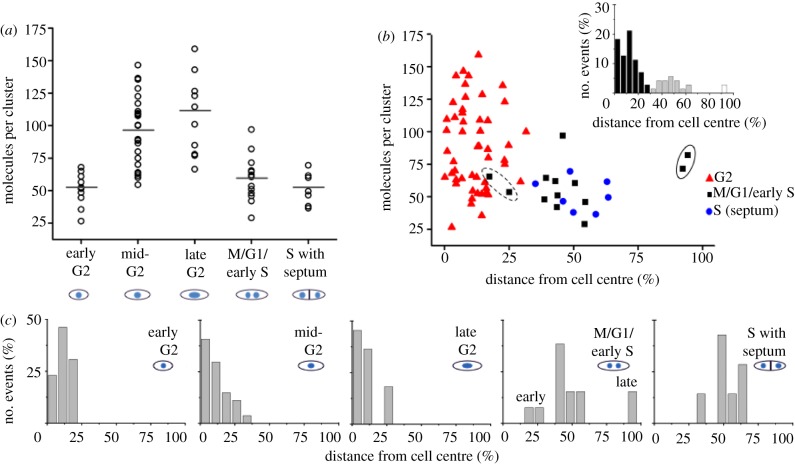
Levels of CENP-A^Cnp1^ molecules peak during late G2. (*a*) Plot of mEos2–CENP-A^Cnp1^ molecules during the cell cycle (strain DL56): early G2 (up to 10.5 µm), mid-G2 (10.5–13.5 µm), late G2 (13.5 µm–mitosis), M/G1/early S and S phase. The horizontal bars indicate the mean value. (*b*) Scatter plot of the number of mEos2–CENP-A^Cnp1^ molecules verses relative distance from the centre of the cell (an indication of progression through metaphase to anaphase). The results from two CENP-A^Cnp1^ clusters from cells in early (dotted) and late (solid) anaphase are circled. The histogram plot (inset) reveals three sub-populations; near the cell centre (black), midzone (grey) and outer/tip (white). (*c*) Expanded histogram plot from (*b*) for each of the cell cycle stages. Early and late anaphase clusters are marked in the M/G1/early S panel.

We next investigated the cell cycle distribution of CENP-A^Cnp1^ molecules in detail by carrying out PALM imaging on individual cells at various stages of the cell cycle. We assigned the localizations found in the centromeric cluster to five different stages of the cell cycle: early G2, mid-G2, late G2, M/G1/early S and S phase (figure 4*a*). All G2 phase cells contained just one cluster of CENP-A^Cnp1^ molecules near the centre of the cell where the nucleus is found (figure 4*b*,*c*). The stage of G2 (early, mid- or late) was determined from the length of the cell. As cells progress through mitosis (M/G1/early S), this single cluster splits into two (early anaphase), which separate and move to the cell poles (late anaphase). Subsequently, each daughter nucleus moves back towards the middle so that they both reside between the mid-zone and the cell tip in the centre of their respective halves of the cell. Cells in early and late anaphase can thus be recognized by the positions of the two clusters of CENP-A^Cnp1^ molecules (figures 1*d*,*e* and 4*b*,*c*). Septum formation occurs towards the end of S phase—thus, S phase cells were identified as those in which the septum had formed prior to cytokinesis and separation of daughter cells.

To simplify the counting of CENP-A^Cnp1^ molecules during dynamic phases of the cell cycle, such as those occurring when chromosomes are separating during mitosis, we used fixed cells for imaging. Fixing the cells did not, however, affect our ability to image and count CENP-A^Cnp1^ molecules because live cells gave the same number of CENP-A^Cnp1^ localizations/molecules (see the electronic supplementary material, figure S8). An analysis of the number of signals found in each centromere cluster showed that cells in early G2 had a relatively stable number of CENP-A^Cnp1^ molecules with an average of 52 per cell (figure 4*a*). As the cells progress through the cell cycle to mid- and then to late G2, the average number of molecules per centromere cluster begins to increase by an average factor of 2 (to 110 molecules) by late G2. This increase in centromeric CENP-A^Cnp1^ levels mirrors that observed in a previous study [9] with GFP-CENP-A^Cnp1^, and our own ChIP–qPCR binding analysis of CENP-A^Cnp1^ during the cell cycle (see the electronic supplementary material, figure S9). Following chromosome segregation, two centromeric clusters are easily detected in each cell. During these stages, which encompass M, G1 and early S phase, the average number of molecules per cluster declines to 59, or around half that found in late G2 cells. This reflects the fact that the six chromosomes and centromeres present in G2 have divided equally between the two daughter cells in mitosis (into two sets of three chromosomes, see the electronic supplementary material, figure S2*b*). As cells complete S phase (DNA replication), and the septum forms in preparation for cell fission, the average number of CENP-A^Cnp1^ molecules per centromere cluster remains at a similar level (52 molecules). Thus, it appears that the replenishment of CENP-A^Cnp1^ levels does not normally occur after duplication of centromeres in S phase as previously suggested [9], even though additionally expressed new CENP-A^Cnp1^ can be deposited in S phase [10]. We suggest that S phase deposition seen by others [9,10] may instead signify CENP-A^Cnp1^ turnover, where old CENP-A^Cnp1^ molecules are being replaced with new CENP-A^Cnp1^ molecules, resulting in no overall change in CENP-A^Cnp1^ protein levels. Finally, cells imaged in both early and late anaphase were found to have similar numbers of CENP-A^Cnp1^ molecules to those analysed in both S phase (septated) and early G2 cells. This rules out the possibility that replenishment occurs during the earlier stages of mitosis (see circled clusters in figure 4*b*). Therefore, unlike metazoan cells, CENP-A^Cnp1^ replenishment at *S. pombe* centromeres does not appear to occur in the late stages of mitosis/early G1 [5–7]. Rather, our data suggest that in *S. pombe* the replenishment of CENP-A^Cnp1^ levels takes place solely in mid- to late G2, prior to the initiation of mitosis. To confirm these results, we repeated the whole experiment (and analysis) using a different fluorophore, PAmCherry1, with very similar results (see the electronic supplementary material, figure S5).

## 4. Conclusions

By studying CENP-A^Cnp 1^ deposition at single-molecule sensitivity using PALM, we were able to quantify the numbers of protein molecules without recourse to comparison with the fluorescence of other proteins or different methods. This approach should therefore be useful for absolute quantification, particularly where the numbers of protein molecules are small and they are densely packed, and not easily detected by other methods. It enabled us to follow CENP-A^Cnp1^ during the period of the cell cycle when the centromeres separate, which in contrast to previous studies [9] allowed us to analyse cells in both the early and late stages of anaphase and show that CENP-A levels are not replenished during S phase. Our finding that CENP-A^Cnp1^ increases at centromeres in G2 suggests a difference to the situation in metazoan cells, where CENP-A deposition occurs in the late stages of mitosis and early G1 phase. However, it is possible that CENP-A^Cnp1^ accumulates at *S. pombe* centromeres during G2 phase (i.e. in preparation for incorporation), but is not actually assembled into nucleosomes until a later stage. The approach that we have demonstrated here should allow this to be investigated in future studies.

Two recent analyses, using comparisons of the fluorescence intensity of CENP-A^Cnp1^ clusters with a variety of other fluorescent standards, gave different estimates for the levels of CENP-A^Cnp1^. In their study, Lawrimore *et al*. [33] concluded that there are approximately 15 CENP-A^Cnp1^ molecules per centromere, a result very consistent with ours, while Coffman *et al*. [31] found that each anaphase cluster contains 680 CENP-A^Cnp1^ molecules (i.e. greater than 200 per centromere). We wondered whether the discrepancy could be due to differences in the way CENP-A^Cnp1^ was tagged, or to other genetic differences in the strains of *S. pombe* employed. Comparison of the CENP-A^Cnp1^ levels using ChIP–qPCR in the different strains employed in previous studies did reveal differences (see the electronic supplementary material, figure S7). In particular, the N-terminally tagged strains constructed by Coffman *et al*. [31] showed significantly lower levels of CENP-A^Cnp1^ than wild-type—indeed, lower levels than our N-terminally tagged mEos2 strain and the GFP-tagged strain constructed by Takayama *et al*. [9]. As with our own N-terminally tagged strains, however, those constructed by Coffman *et al*. [31] did not exhibit higher levels of histone H3 or growth defects, suggesting that CENP-A^Cnp1^ deposition is not affected by the N-terminal tags and that the reduced ChIP signal is due to epitope masking by the fluorophore. This finding is consistent with their fluorescence experiments, which suggest a mean of between approximately 600 and 400 molecules per anaphase cluster for two different N-terminally tagged strains—compared with the mean of 680 for the C-terminally tagged protein [31]. We conclude that N-terminal tagging with a fluorescent protein does not substantially affect CENP-A^Cnp1^ deposition.

At present, it is not clear why the approach employed by Coffman *et al*. [31] gave much higher estimates for the number of CENP-A^Cnp1^ localized to the centromere than our own. However, it is difficult to reconcile how greater than 100 nucleosomes/centromere suggested by their results, which is fivefold more than our maximum estimate, could be deposited in the approximately 10 kb region of each centromere. In particular, in the recent crystal structure of the CENP-A^Cnp1^ nucleosome [23], around 120 bp of DNA is condensed, suggesting that only approximately 80 nucleosomes could assemble at a centromere if there was no linker DNA at all.

Regardless of the precise number of CENP-A^Cnp1^ nucleosomes present at the centromeres in *S. pombe*, our demonstration of the feasibility of imaging proteins at the single-molecule level, and studying the relative numbers involved in a particular process through the cell cycle, provides a powerful demonstration of how this method will enhance future studies of CENP-A deposition and other chromatin-based processes. In particular, the recent development of novel fluorophores will in future allow both pulse-chase and two-colour PALM co-localization studies [34]. Within live cells, it should now be possible to more precisely investigate in a biological context the assembly/disassembly of multi-protein complexes that play a key role in the organization of chromatin to control genetic and epigenetic processes, such as those associated with the epigenetic inheritance of CENP-A.

## 5. Material and methods

### 5.1. Yeast strains

The genotypes of the strains used in this study are listed in the electronic supplementary material, table S1. Standard methods and techniques for fission yeast genetic manipulations were employed. A two-step process was used to N-terminally tag the endogenous Cnp1 locus with either mEos2 or PAmCherry1. Firstly, a PCR-amplified cassette containing the ura4:nmt41 fragment from the pAW33 plasmid [35] was incorporated by homologous recombination at the N-terminal Cnp1 locus. The ura4:nmt41 insertion was then replaced by a homologous recombination with a fragment containing either the mEos2 [14] or PAmCherry1 cDNA [20], to generate the N-terminally tagged mEos2 (DL56) and PAmCherry1 (DL70) Cnp1 strains containing the native and intact Cnp1 promoter. The pREPnmt81mEos2 plasmid containing the mEos2 gene was constructed by inserting the mEos2 fragment generated by PCR from pRSETamEos2 [14] into pREPNT81 [36]. All cloning and strains were verified by DNA sequencing.

### 5.2. Sample preparation for photo-activated localization microscopy imaging

All strains used for imaging were grown at 30°C for six to eight cell divisions to mid log phase (OD_600_ 0.5–1.0) in Edinburgh minimal medium (EMM) (containing 200 mg l^−1^ amino acids as required). To vary the expression levels of the mEos2 fluorophore, thiamine (5, 10, 25 µg ml^−1^) was added to the media of strains carrying the nmt81mEos2 plasmid. For fixed cell samples, cells (2.0 × 10^7^) were resuspended in 1 ml of phosphate buffer (100 mM sodium phosphate pH 7.5) containing 1 per cent paraformaldehyde and incubated at room temperature for 20 min. Fixed cells were washed (three times) with 1 ml of phosphate buffer, and the pellet resuspended in 400 µl of phosphate buffer. Then, 200 µl of fixed cells was added to a single well of a Labtek 8/well glass chamber slide (Nunc). Before use, Labtek slides were cleaned with 0.5 per cent hydrofluoric acid and then coated with polylysine (Sigma). After a 10 min incubation at room temperature, non-adherent cells were washed away with phosphate buffer. To prevent cells from shaking/wobbling, adherent cells were briefly fixed to the polylysine-coated slide with 1 per cent paraformaldehyde/phosphate buffer for 5 min at room temperature. For imaging, cells were overlayed with phosphate-buffered solution. For live cell samples, cells (1.0 × 10^7^) were mixed with 1 ml of 0.5 per cent low melting point agarose (Sigma) containing EMM media (with 200 mg l^−1^ amino acids as required). Then, 200 μl of the cell/agarose/EMM mix was overlayed onto a single well of a Labtek 8/well glass chamber slide (Nunc). Before use, Labtek slides were cleaned with 0.5 per cent hydrofluoric acid. Once set, 200 µl of EMM was added to each well to prevent the agarose pads from drying out. All live-cell imaging was carried out at room temperature.

### 5.3. Photo-activated localization microscopy microscope

PALM experiments were performed on a custom-built microscope described previously [37]. A multi-line argon–krypton laser (Innova 70C, Coherent, USA) and a diode laser emitting at 378 or 405 nm (Cube, Coherent, USA) were coupled to an inverted microscope (IX71, Olympus, Japan) equipped with a 60× oil immersion objective (PlanApo 60×, NA 1.45, Olympus) that results in a depth of field value of approximately 800 nm. Excitation light and fluorescence light were separated using a dichroic mirror (FF410/504/588/669-Di01, AHF, Germany), and appropriate filters were placed in the detection path (568LP and BP610/75, AHF, Germany). The fluorescence signal was recorded with an electron-multiplying CCD camera (EMCCD; Andor Ixon DU897, Belfast, UK). The spot density was kept below 0.1 per frame to avoid multiple-spot events that would lead to quantification errors (see the electronic supplementary material, figure S3*d*) [38]. Raw data were processed using the rapidSTORM software [39]. A detailed description of the single-molecule counting analysis is described in the electronic supplementary material, section ‘Material and methods’.

### 5.4. CENP-A^Cnp1^ nucleosome mapping

ChIP was performed as described [40] with the following modifications. *S. pombe* strains were grown in complete media (YES) at 32°C to 5 × 10^6^ cells ml^−1^, fixed for 15 min in 1 per cent formaldehyde (Sigma) and treated with 0.4 mg ml^−1^ Zymolase (AMS Biotechnology Europe) in PEMS for 1 h. To fragment the DNA, 300 µl aliquots of sample was either sonicated for 20 min in a Bioruptor (Diagenode) or treated with 0.4 U miccrococcal nuclease (MNase; Sigma) at 37°C for 6 min in MNase digestion buffer (50 mM Hepes pH 7.5, 50 mM NaCl, 5 mM MgCl_2_, 1 mM CaCl_2_, 1× Proteinase inhibitor mix (Sigma) and 1 mM phenylmethylsulfonyl fluoride (PMSF)). The MNase reaction was stopped by adding 300 µl lysis buffer 2 (50 mM Hepes pH 7.5, 255 mM NaCl, 12 mM EDTA, 2% Triton X-100, 0.2% sodium deoxycholate, 1× Proteinase inhibitor mix and 1 mM PMSF). Samples were electrophoresed on a 1.5 per cent agarose gel to check the MNase digestion. The samples were incubated for 4–5 h with 10 µl α-Cnp1-antiserum [41] and 40 µl Protein G agarose beads (Roche). The beads were washed as described and incubated with 1 per cent SDS overnight at 65°C. The supernatant was purified using a PCR purification kit (Qiagen). Solexa/Illumina libraries were then prepared as described in the manufacturer’s hand book (Illumina) with the following modifications: bar-coded linkers were used for ligation (see the electronic supplementary material, table S2), and the 150 ± 50 bp library fraction was extracted from an agarose gel to obtain mainly nucleosomal-sized DNA fragments. These fragments were sequenced using an Illumina GAII sequencer (The Genepool, Edinburgh, UK). To map ChIP-seq data, Fastq files were mapped onto the *S. pombe* genome assembly RefSeq NC_003424.1, NC_003424.2, NC_003424.3 (Université de Montreal, Canada) using Novoalign (www.novocraft.com). Mapped reads were divided by the number of possible mapping events per sequencing fragment (which accounts for repeated elements in the genome). From the MNase-digested samples, paired-end reads were mapped (a total of 810 000 paired reads). From the sonicated samples, 2 300 000 single-end reads were mapped with the assumption that the library fragments were on average 150 bp. The number of reads in all samples were normalized to the 810 000 paired-end reads. Biological replicates of both sequencing experiments were performed, and a total of 1 100 000 or 860 000 reads for MNase digestion or sonication were mapped, respectively.

## 6. Acknowledgements

We thank S. McKinney for the mEos2 and V. V. Verkhusha for the PAmCherry1 constructs. This work was funded by a doctoral training programme from the Engineering and Physical Science Research Council (UK) to P.D.D., Marie Curie International Incoming Fellowship (IIF27580) to L.S., Medical Research Council (UK; G0600223) and European Research Council (268788-SMI-DDR) grants to A.M.C., the German Ministry of Education and Research (BMBF, grant nos. 0315262 and 13N9234) to M.H and M.S. and the Wellcome Trust (065061: R.C.A.; 092076: core funding for the Centre for Cell Biology; 082010: E.D.L.).

## Supplementary Material

Supplementary methods, figures and tables
