# Supplementary material for: Quantitative single-molecule microscopy reveals that CENP-ACnp1 deposition occurs during G2 in fission yeast
Source: Open Biol. 2012 Jul;2(7):120078. doi: 10.1098/rsob.120078 (PMC3411111; doi:10.1098/rsob.120078)
Supplement: Supplementary methods, figures and tables [file rsob120078-s1.pdf]

Supporting Information

Material and methods

Data analysis

A) Single-molecule counting analysis

Single-molecule movie files were analyzed with the rapidSTORM software package (34) using an intensity threshold of 600 photons (Supplementary Figure S3A). In the first step, the software provides coordinates of single fluorophores. In the second step, the software groups localizations of a single fluorophore that were recorded in sequential imaging frames by Kalman-filtering, which accounts for 'on-times' of different length. A cut-off distance of 40 nm, which represents twice the experimental resolution (see Supplementary Figure S3C), was chosen as the initial parameter for the Kalman filter. We calibrated this approach with data recorded from *S. pombe* expressing low amounts of mEos2 in the cytosol and determined that a single mEos2 fluorophore generates on average 1.98 localizations due to blinking (see Figure 2). Notably, mEos2 appeared randomly distributed within the cells and showed no apparent clustering (see Figures 2A-C).

The ability to detect proteins in single-molecule PALM imaging depends on various experimental factors. We assume that all molecules of mEos2-CENP-A^{Cnp1} carry a functional fluorescent protein and that the photo-conversion of mEos2 is almost quantitative (*13*). However, we cannot exclude photo-activation or photo-bleaching of a subset of mEos2 prior to imaging, although care was taken to minimize the exposure of the samples to light.

During the imaging experiment, we applied low intensity UV laser irradiation to avoid photo-activation of more than one fluorophore per diffraction-limited area. In the later stages of an experiment, we made sure that we had photo-activated and recorded all the mEos2 fluorophores in a cell by gradually increasing the UV laser intensity and imaging for a sufficiently long time (Supplementary Figure S3D).

In control experiments with wild type *S. pombe*, lacking any mEos2 protein, we determined that the number of background single-molecule localizations per square micron was less than

2 (Supplementary Figure S3B), which translates to a contribution of less than 0.5 single-molecule localizations per CENP-A^{Cnp1} cluster (assuming a maximum area of 0.5 x 0.5 μm^2 observed in our experiments). We also determined that the number of cellular mEos2-CENP-A^{Cnp1} single-molecule localizations per square micron (outside the clusters) was less than 3 (Supplementary Figure S3E), which contributes less than 0.75 localizations per cluster.

B) Post data image analysis

All calculations and analysis of cell length, cluster position, number of localizations/molecules etc., were carried out using ImageJ, Origin (OriginLab) and Microsoft Excel software.

Cell cycle experiments

Cell cycle block-release was performed using a temperature sensitive *cdc25-22* mutant. Cells were grown in complete media (YES) at (permissive temperature) and the cell cycle block at G2/M phase was imposed by incubating the culture at 36°C (restrictive temperature) for 3 hours. This block was released by shifting the culture to 25°C. Samples were taken at 20 minute intervals, and the septation index was determined prior to fixation with 1% formaldehyde. Samples were then prepared for ChIP.

Chromatin Immunoprecipitation (ChIP) experiments to compare tagged Cnp1 strains

10μl α-Cnp1-antiserum [37] was used to perform Cnp1 ChIPs. For histone H3 ChIPs, 2μl rabbit polyclonal antibody to histone H3 (Abcam ab1791) was used.

Quantitative Real-Time PCR

Quantitative PCR (qPCR) was performed using a LightCycler® 480 Real-Time PCR System (Roche) according to the manufacturers instructions with the LightCycler® 480 SYBR Green I Master mix (Roche). Primers sequences are described in Supplementary Table S2. All measurements were replicated 2-4 times.

Supplementary Figures

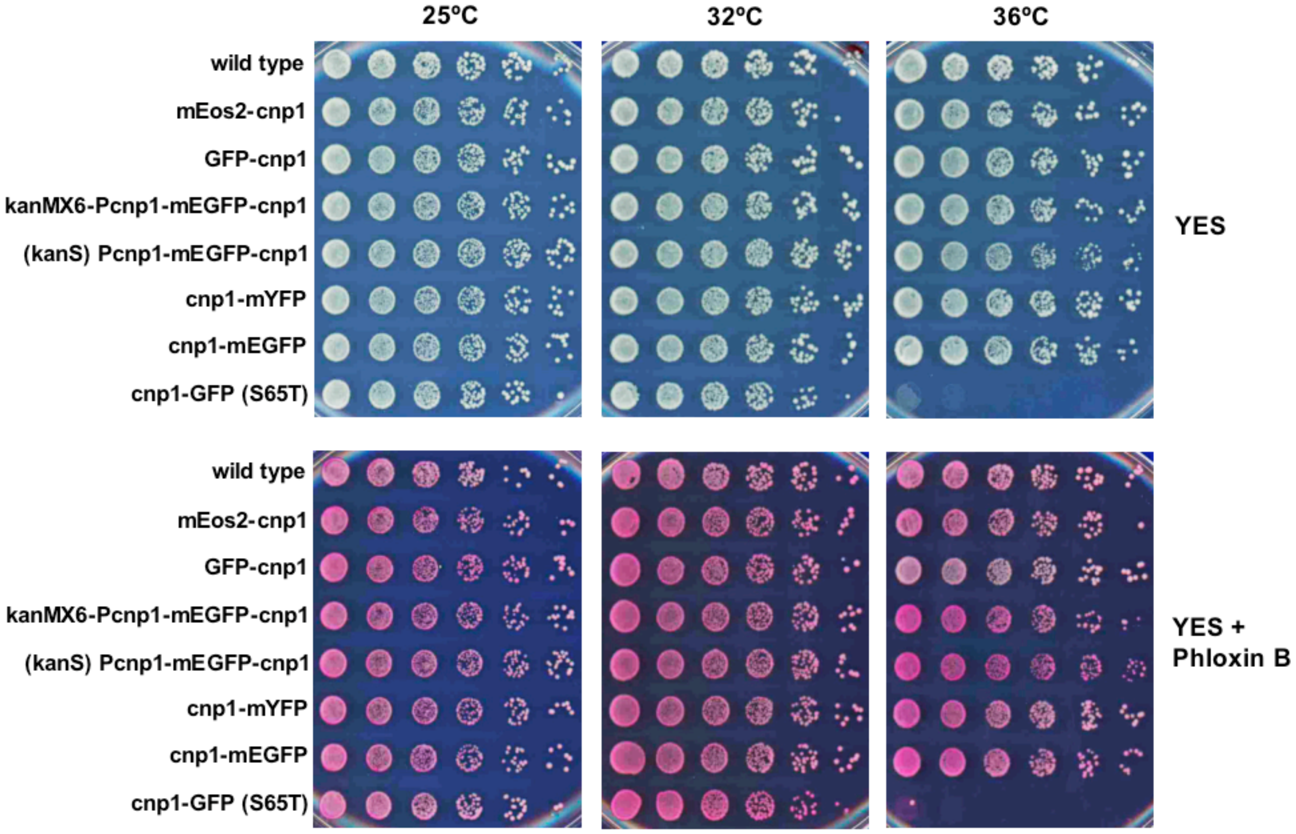

Supplementary Figure S1: Growth test of yeast strains

Growth analysis of mEos2-CENP-A^{Cnp1} and various other GFP tagged CENP-A^{Cnp1} strains. Cultures of the indicated strains were serially diluted and spotted onto YES plates (+/- Phloxin B 2.5μg/ml) and grown at various temperatures as indicated. Strain numbers: wild type (1645); mEos2-Cnp1 (DL56); GFP-cnp1 (sp/KT1769), Takayama et. al. [8]; and kanMX6-Pcnp1-mEGFP-cnp1 (JW2595), (kanS) Pcnp1-mEGFP-cnp1 (JW2648), cnp1-mYFP (JW1469), cnp1-mEGFP (JW1470) and cnp1-GFP (S65T) (JW3523), Coffman et. al. [28].

Note that the C-terminal tagged cnp1-GFP (S65T) strain displays slower growth at elevated temperatures, as previously reported [28].

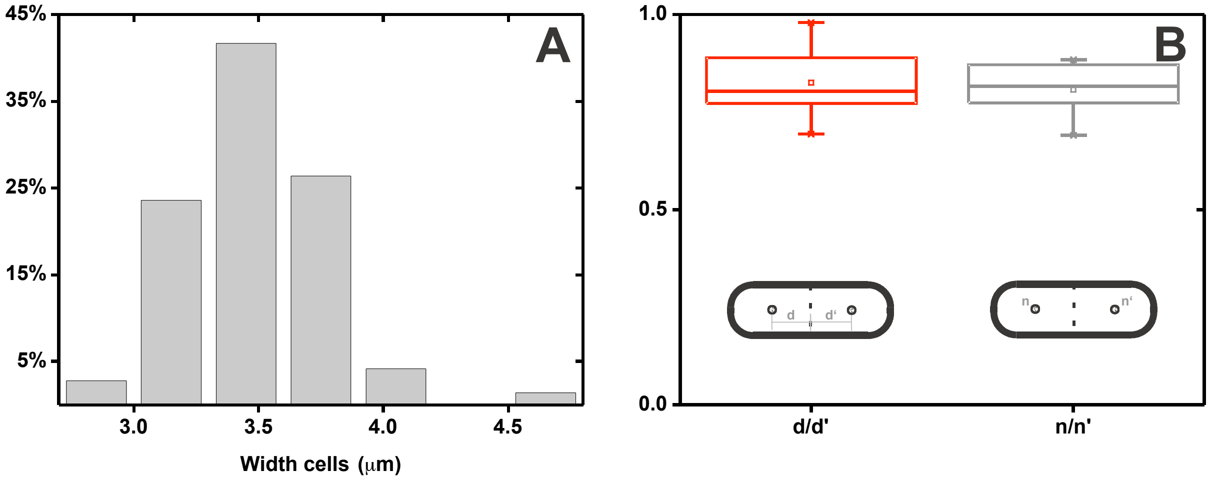

Supplementary Figure S2: Parameters to identify healthy cells.

A. We used the width of *S. pombe* cells as one measure to identify healthy cells. For example, among 65 cells, we excluded one cell from the data analysis which was abnormally wide (>4.5 μm).

B. Analysis of both the distance (d) of CENP-A^{Cnp1} clusters (d/d') from the centre of the cell, as well as the number (n) of CENP-A^{Cnp1} molecules per cluster (n/n') in mitotic, G1 and S phase cells reveal that CENP-A^{Cnp1} clusters are highly symmetric. This indicates that there are no-lagging chromosomes in the cells analysed and that all segregate their chromosomes equally.

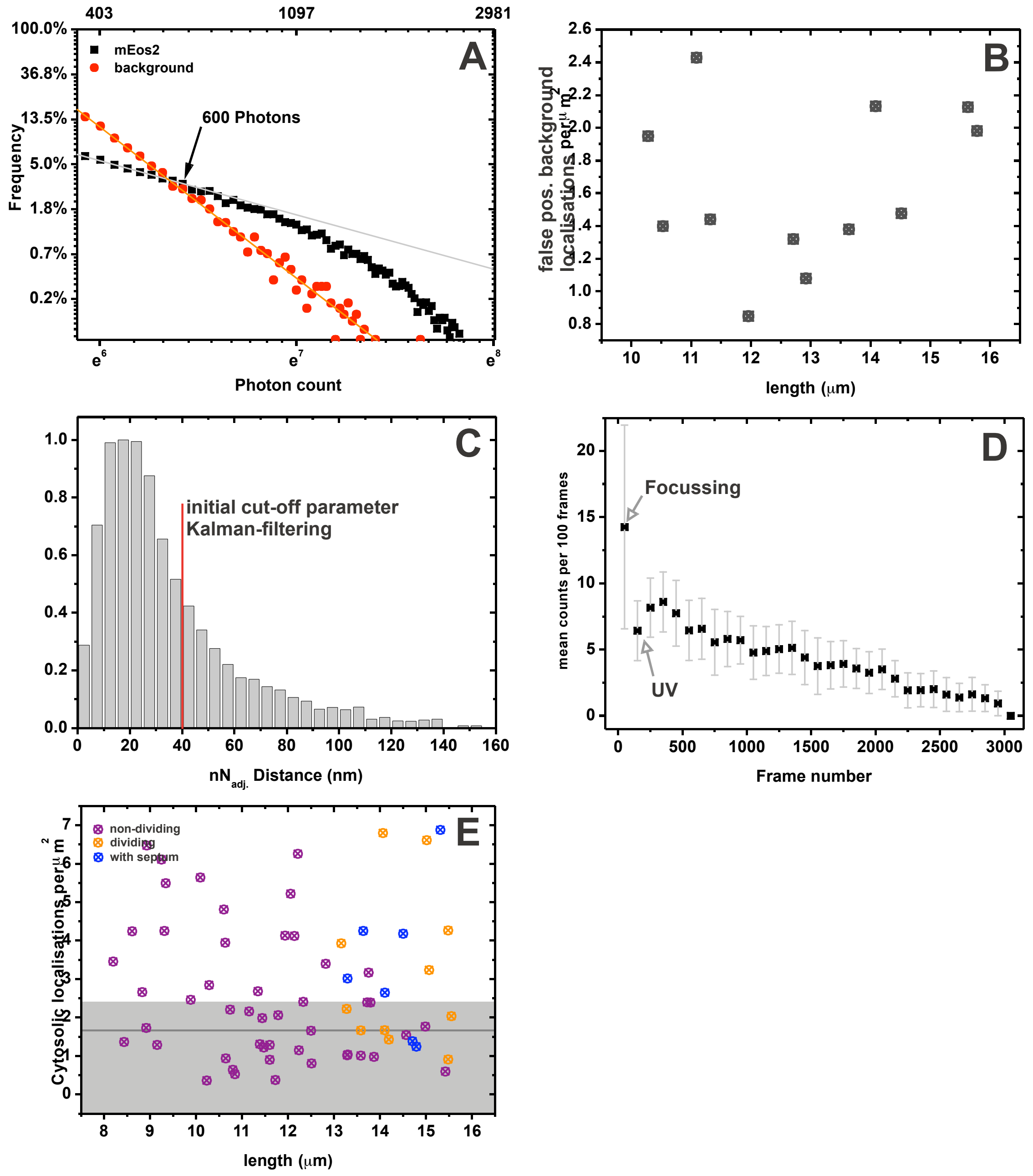

Supplementary Figure S3: Experimental PALM-resolution and localization (spot) density

A. Derivation of the photon threshold for single-molecule localization analysis. Background counts recorded from wild-type cells (BG0000H6) without mEos2 (red) show mono-exponential behavior, whereas cells expressing mEos2-CENP-A^{Cnp1} deviate from mono-exponential behavior because the fluorophore has Gaussian photon statistics. From these curves, we determined a background intensity threshold of 600 photons.

B. False-positive single-molecule localizations recorded for the wild-type (BG0000H6) strain (photon threshold 600). On average, 1.63 localizations per μm^2 were found which translates to less than 0.5 false-positive single-molecule localizations per CENP-A^{Cnp1} cluster (assuming a cluster area of 0.5 x 0.5 μm^2).

C. Nearest neighbour distances of mEos2-localizations in adjacent frames. Most of the localizations in adjacent frames, taking into account the low spot density (D), are caused by fluorescence of the same molecule. Using this, an experimental resolution for the setup of 21nm can be extracted, which is close to the theoretical value of <15nm. For quantification, so as not to overcount the molecules, the start value of the Kalman filtering (tracking filter) was therefore set to 40 nm as described in the materials and methods, getting more precise the longer the molecule trace was.

D. The plot shows the number of single-molecule localizations in a PALM experiment over time (binned to 100 frames). The initial spot density was about 0.14 spots/frame and decreases to 0 when all the mEos2 fluorophores have been detected.

E. Number of mEos2- CENP-A^{Cnp1} molecules (not part of a cluster) in *S. pombe*. A maximum number of 7 localizations were found per μm^2, which translates to about 2.7 single-molecule localizations per CENP-A^{Cnp1} cluster (assuming a cluster area of 0.5 x 0.5 μm^2). No correlation with the cell cycle stage can be observed. The grey area indicates the average false-positive localizations (or background localizations found in a non mEos2 tagged strain, see B above).

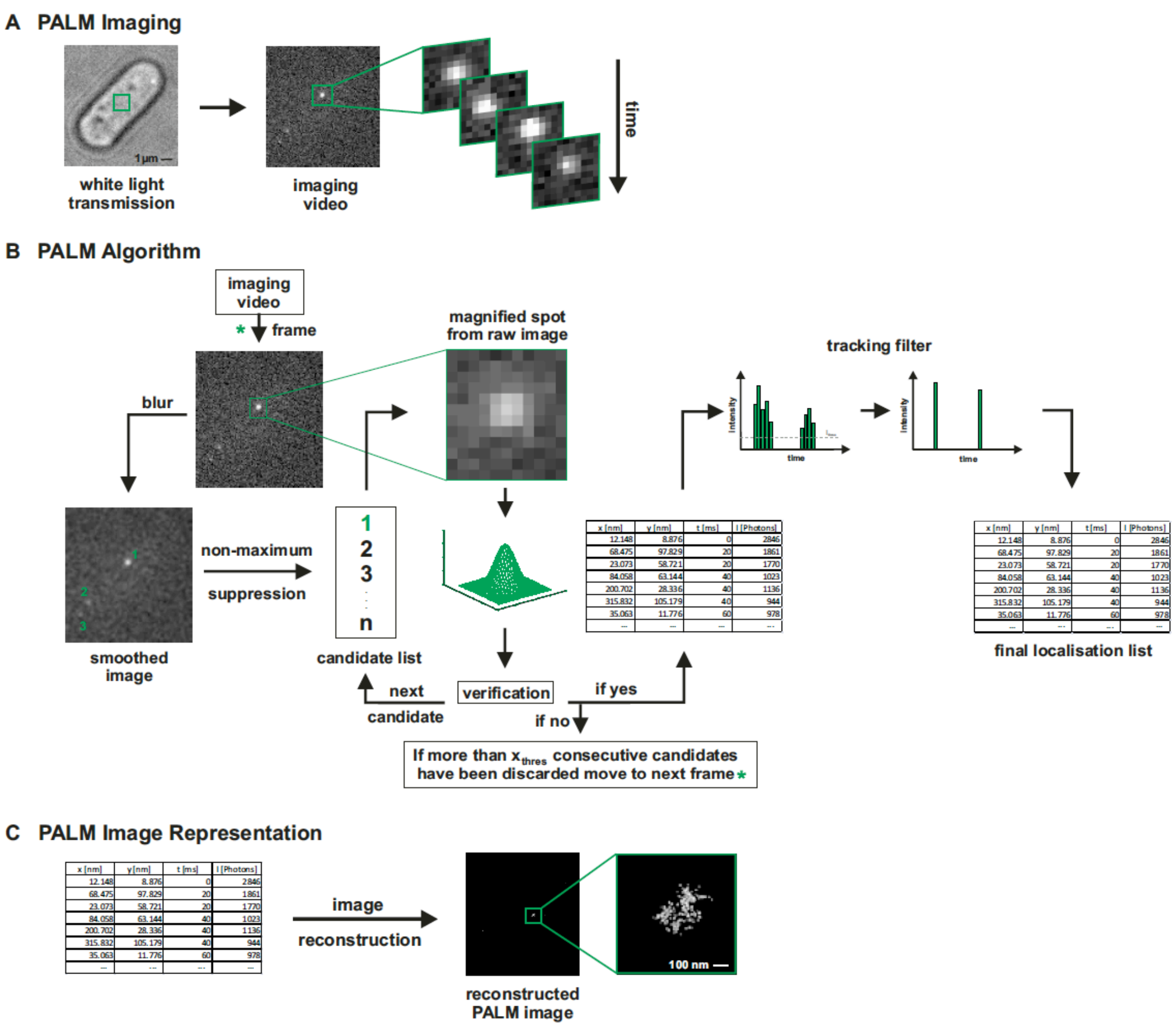

Supplementary Figure S4: PALM imaging

(A) Individual cells to be imaged are identified by white light transmission. The entire cell is then imaged under PALM conditions (see material and methods), where individual frames containing single molecule events are captured over time, with a frame rate of 100ms, to generate a video movie.

(B) Schematic of algorithm used to identify single molecule localizations in a PALM video. To identify single molecule localizations each frame of the imaging movie is analysed separately. Initially, the frame is smoothed and the localization candidates are identified by a non-maximum suppression routine, then stored in a candidate list, which is sorted by photon intensity (1,2,3...n). Candidates (starting from 1) are then fitted using the original, non-blurred

image with a Gaussian. The fit is checked by verification parameters (intensity threshold, symmetry of gaussian, sigma etc.). If accepted, the fit is stored in a localization list and the next candidate is analysed using this fitting and verification routine. As low intensity candidates are more likely to fail the routine (35), we apply a x_{thres} step to the routine. If more than x_{thres} (normally set to 10) consecutive candidates do not pass the fit and verification routine, the remaining candidates on the list (containing lower intensities) are discarded and the next frame is analysed. Once all the frames of a movie are analysed a tracking filter is applied to the localization list, to remove localizations from single molecule fluorescent events captured in several consecutive frames. The tracking filter then creates a final list in which one localization equals one fluorescent event.

(C) The parameters from the final list are then used to reconstruct the PALM image. Fit centres for each localization are plotted onto an image with a pixel size value of 10x10nm. To distinguish single localizations within one pixel from multiple localizations the plotted fit centres are histogrammed accordingly.

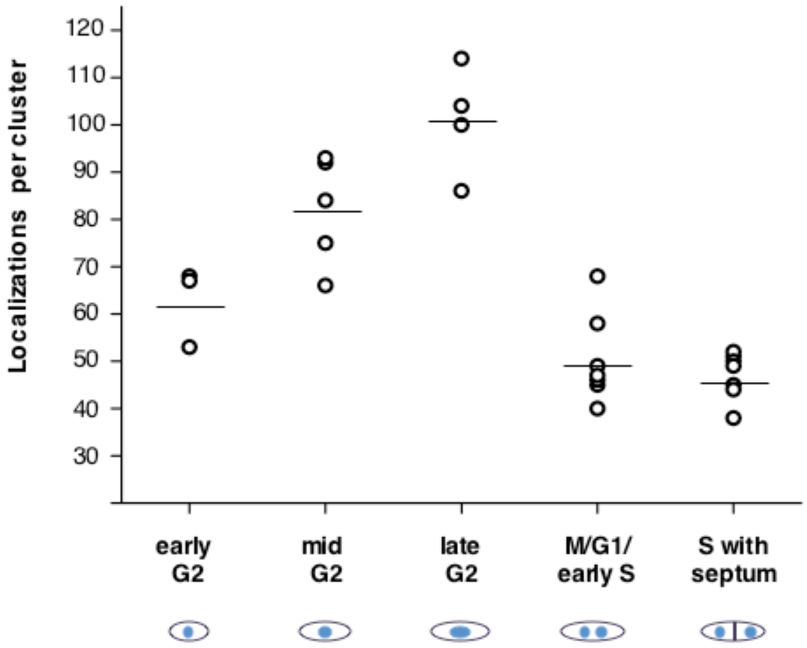

Supplementary Figure S5: PALM analysis of PAmCherry1-CENP-A^{Cnp1} during the cell cycle

Plot of PAmcherry1-CENP-A^{Cnp1} localizations during the cell cycle. Early G2 (up to 10.5 μm), mid G2 (10.5-13.5 μm), late G2 (13.5 μm-mitosis), M/G1/early S, and S phase (with septum). The horizontal bars indicate mean values.

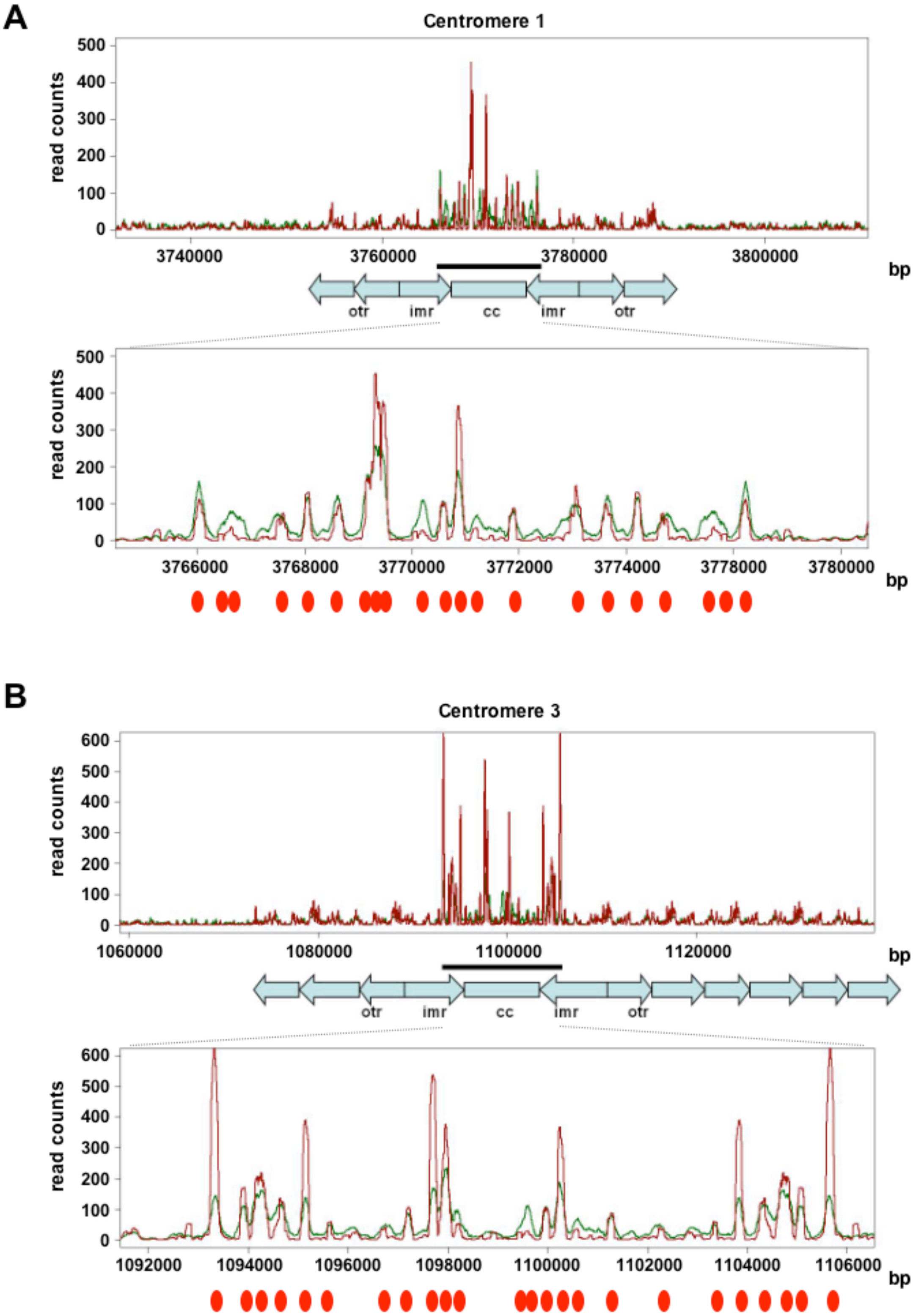

Supplementary Figure S6: Distribution of CENP-A^{Cnp1} nucleosomes across the centromere of chromosomes 1 and 3

Genome browser view of the centromere region from chromosome 1 (A) and chromosome 3 (B), showing ChIP-seq profiles of CENP-A^{Cnp1} from sonicated (green) and MNase digested samples (red). Shown below each plot is an expanded view of the central kinetochore domain (black bar) for each centromere. Data on the y axis are normalised read counts, and the x axis is genome position on each chromosome in base pairs. Red ovals indicate the positions of CENP-A^{Cnp1} nucleosomes.

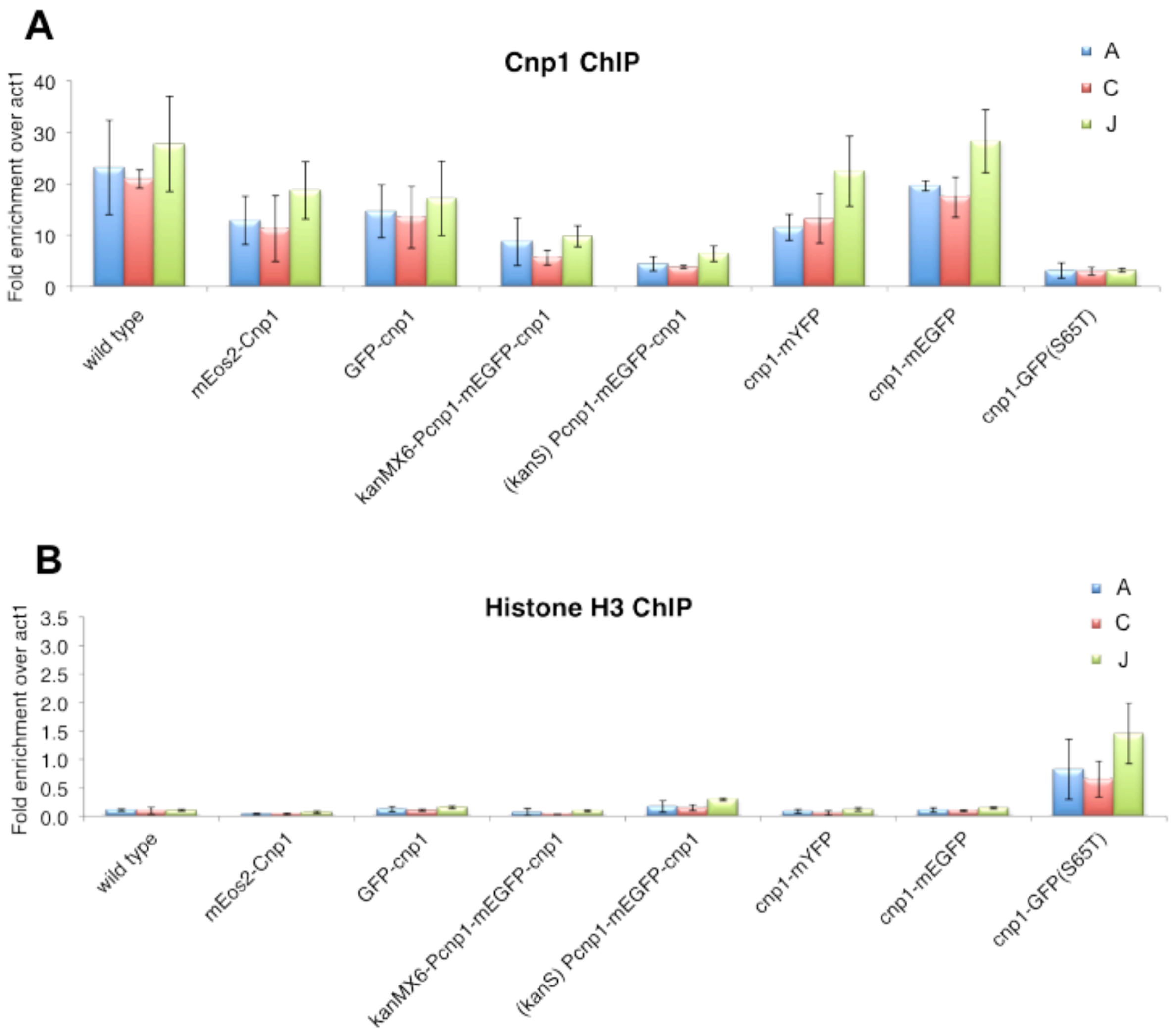

Supplementary Figure S7: Comparision of CENP-A^{Cnp1} and histone H3 levels in mEos2-CENP-A^{Cnp1} with other GFP tagged Cnp1 strains

ChIP-qPCR of CENP-A^{Cnp1} (A) and histone H3 (B) at three different locations within the central domain of chromosome 2 (C, A and J; see Figure 2). Error bars indicate standard deviations determined from 3 ChIP replicates. Strain numbers: wild type (1645); mEos2-Cnp1 (DL56); GFP-cnp1 (sp/KT1769), Takayama et. al. [8]; kanMX6-Pcnp1-mEGFP-cnp1 (JW2595), (kanS) Pcnp1-mEGFP-cnp1 (JW2648), cnp1-mYFP (JW1469), cnp1-mEGFP (JW1470) and cnp1-GFP (S65T) (JW3523), Coffman et. al. [28].

Note that the cnp1-GFP (S65T) strain that displayed a slow growth phenotype (Supplementary Figure S1) has significantly increased levels of H3 at these 3 loci, due to the levels of CENP-A^{Cnp1} being greatly reduced.

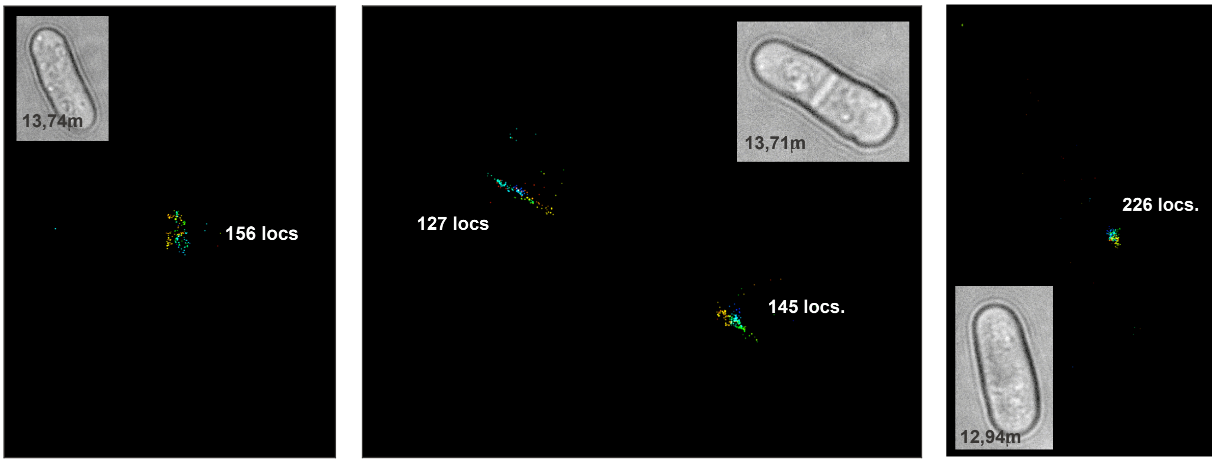

Supplementary Figure S8: Live-cell mEos2-CENP-A^{Cnp1} imaging

Live *S. pombe* cells mounted in agarose pads were imaged with PALM. Image analysis was performed using the same parameters as for the fixed samples. Single-molecule localizations were color-coded according to the frame number (red to blue) to visualize the movement of the CENP-A^{Cnp1} clusters.

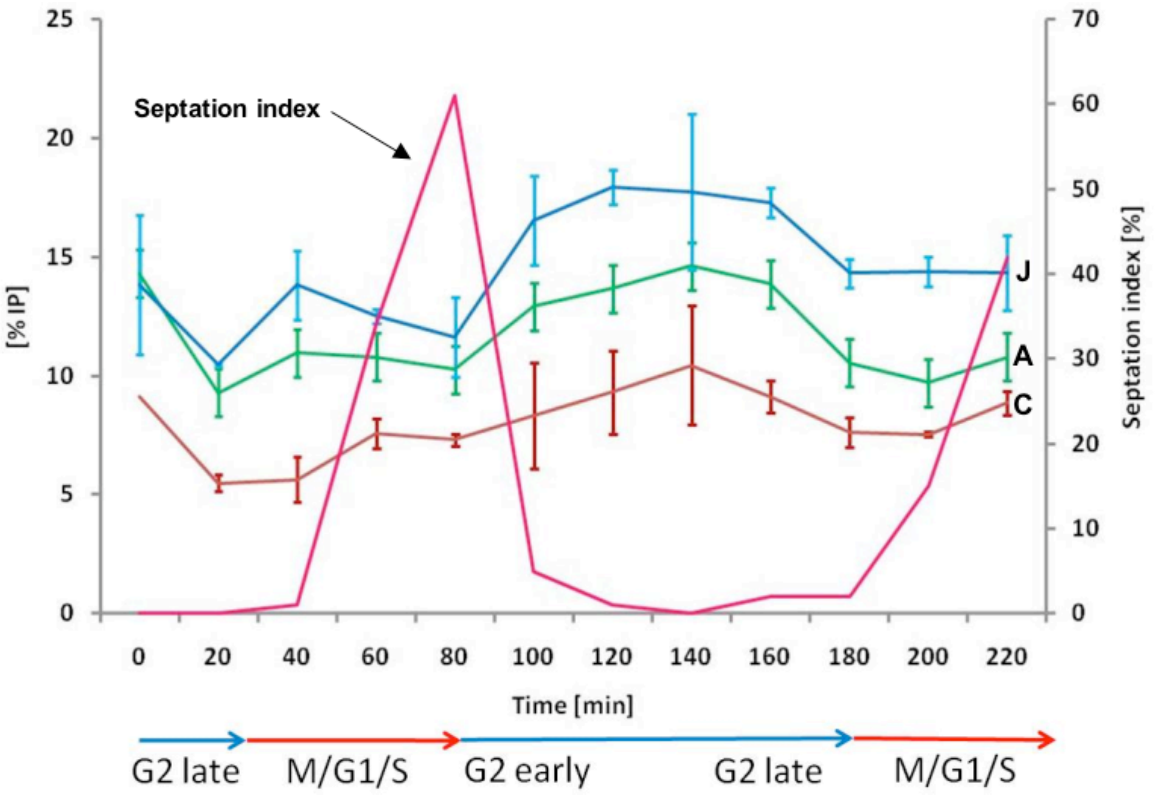

Supplementary Figure S9. **CENP-A^{Cnp1} association at centromere 2 during the cell cycle**

qPCR of CENP-A^{Cnp1} ChIP at three different locations (C, A and J; see Figure 2) within the central domain of chromosome 2 at 20 minute time points following release of G2 blocked *cdc25-22* cells (36ºC) into the cell cycle by shifting to the permissive temperature (25ºC). The percentage of septated cells was counted at each time point to monitor cell cycle progression and the level of sychrony (Septation index in magenta). Error bars indicate standard deviations from ChIP replicates.

Supplementary Table S1 *S. pombe* strains used in this study

Strain	Genotype	Source
BG0000H6	*h^+, ade6-210, leu1-32, ura4-D18*	Bioneer
DL56	*h^+, ade6-210, leu1-32, ura4-D18, mEos2:cnp1*	This study
DL70	*h^+, ade6-210, leu1-32, ura4-D18, PAmCherry1:cnp1*	This study
DL 41	*h^+, ade6-210, leu1-32, ura4-D18, pREPnmt81-mEos2*	This study
143 (972)	*h^-*	Allshire Lab
151	*h^-, leu1-32, cdc25-22*	Allshire Lab
1645	*h^+, ade6-210, arg3-D4, his3-D1, leu1-32, ura4-D18*	Allshire Lab
sp/KT1769	*h^-, leu1-32, ura4-D18, lys1, GFP-cnp1-NAT*	8
JW2595	*h^-, ade6-M210, leu1-32, ura4-D18, kanMX6-Pcnp1-mEGFP-cnp1*	28
JW2648	*$h^?$, ade6-M210, leu1-32, ura4-D18, (kanS) Pcnp1-mEGFP-cnp1*	28
JW1469	*h^-, ade6-M210, leu1-32, ura4-D18, cnp1-mYFP-kanMX6*	28
JW1470	*h^-, ade6-M210, leu1-32, ura4-D18, cnp1-mEGFP-kanMX6*	28
JW3523	*h^-, ade6-M210, leu1-32, ura4-D18, cnp1-GFP(S65T)-kanMX6*	28

Supplementary Table S2 Primers used for Solexa and qPCR analysis. * indicates a Phosphorothioate link, [Phos] indicates a 5’-phosphorylated primer, bold letters indicate barcodes.

HB420	GACGTAATCTAGGACCCCTACG	a-fragment, qPCR
HB421	GCAAGGGAGAGGCAATACAG	a-fragment, qPCR
HB424	AAGCGCTAACTCGTTTAAGTGAA	c-fragment, qPCR
HB425	ACATGGCGTGGAAAGTCATC	c-fragment, qPCR
HB438	TTTCAAAGAAAACAGCCAAAGG	j-fragment, qPCR
HB439	TCAGACAATTAAAGCAGTTGACG	j-fragment, qPCR
HB190	ACACTCTTTCCCTACACGACGCTCTTCCGATCT**GG*T**	Linker GGT
HB192	ACACTCTTTCCCTACACGACGCTCTTCCGATCT**AA*T**	Linker AAT
HB194	ACACTCTTTCCCTACACGACGCTCTTCCGATCT**CG*T**	Linker CGT
HB196	ACACTCTTTCCCTACACGACGCTCTTCCGATCT**AT*T**	Linker ATT
HB214	[Phos]**CC**AGATCGGAAGAGCGGTTCAGCAGGAATGCCGAG	Linker GGT
HB215	[Phos]**TT**AGATCGGAAGAGCGGTTCAGCAGGAATGCCGAG	Linker AAT
HB216	[Phos]**CG**AGATCGGAAGAGCGGTTCAGCAGGAATGCCGAG	Linker CGT
HB217	[Phos]**AT**AGATCGGAAGAGCGGTTCAGCAGGAATGCCGAG	Linker ATT
HB162	CAAGCAGAAGACGGCATACGAGATCGGTCTCGGCATTCCT GCTGAACCGCTCTTCCGATC*T	Solexa Library PCR primer 1.1
HB163	AATGATACGGCGACCACCGAGATCTACACTCTTTCCCTAC ACGACGCTCTTCCGATC*T	Solexa Library PCR primer 2.1
HB475	CCGGCGAGATCAAGACGCAT	act1, qPCR
HB476	TATGTTGCTATTCAAGCTG	act1, qPCR
